# Advancing microbiome research through standardized data and metadata collection: introducing the Microbiome Research Data Toolkit

**DOI:** 10.1093/database/baae062

**Published:** 2024-08-21

**Authors:** Lyndon Zass, Lamech M Mwapagha, Adetola F Louis-Jacques, Imane Allali, Julius Mulindwa, Anmol Kiran, Mariem Hanachi, Oussama Souiai, Nicola Mulder, Ovokeraye H Oduaran

**Affiliations:** Computational Biology Division, Department of Integrative Biomedical Sciences, IDM, University of Cape Town, Rondebosch, Cape Town 7701, South Africa; Department of Biology, Chemistry and Physics, Faculty of Health, Natural Resources and Applied Sciences, Namibia University of Science and Technology, Private Bag 13388, 13 Jackson Kaujeua Street, Windhoek, Namibia; Department of Obstetrics and Gynecology, Division of Maternal-Fetal Medicine, University of Florida, 1600 SW Archer Road, Gainesville, FL 32610, USA; Laboratory of Human Pathologies Biology, Department of Biology, Faculty of Sciences, Mohammed V University in Rabat, Rabat, Morocco; Department of Biochemistry and Sports Sciences, College of Natural Sciences, Makerere University, P.O. Box 7062, Kampala, Uganda; Malawi-Liverpool-Wellcome Trust, P.O. Box 30096, Blantyre 3, Malawi; Institute of Infection, Veterinary and Ecological Sciences, University of Liverpool, Liverpool CH64 7TE, UK; Laboratory of Bioinformatics, Biomathematics and Biostatistics (LR16IPT09), Institute Pasteur of Tunis, University Tunis El Manar, 13, Place Pasteur, B.P. 74, Tunis 1002, Tunisia; Laboratory of Bioinformatics, Biomathematics and Biostatistics (LR16IPT09), Institute Pasteur of Tunis, University Tunis El Manar, 13, Place Pasteur, B.P. 74, Tunis 1002, Tunisia; Computational Biology Division, Department of Integrative Biomedical Sciences, IDM, University of Cape Town, Rondebosch, Cape Town 7701, South Africa; Sydney Brenner Institute for Molecular Bioscience, University of the Witwatersrand, 9 Jubilee Road, Parktown 2193, Johannesburg, Johannesburg, South Africa

## Abstract

Microbiome research has made significant gains with the evolution of sequencing technologies. Ensuring comparability between studies and enhancing the findability, accessibility, interoperability and reproducibility of microbiome data are crucial for maximizing the value of this growing body of research. Addressing the challenges of standardized metadata reporting, collection and curation, the Microbiome Working Group of the Human Hereditary and Health in Africa (H3Africa) consortium aimed to develop a comprehensive solution. In this paper, we present the Microbiome Research Data Toolkit, a versatile tool designed to standardize microbiome research metadata, facilitate MIxS-MIMS and PhenX reporting, standardize prospective collection of participant biological and lifestyle data, and retrospectively harmonize such data. This toolkit enables past, present and future microbiome research endeavors to collaborate effectively, fostering novel collaborations and accelerating knowledge discovery in the field.

**Database URL**: https://doi.org/10.25375/uct.24218999.v2

## Introduction

The field of microbiome research has seen a significant growth due to advancements in next-generation sequencing technologies. The microbiome, which plays a vital role in various biological processes and its impact on human health, has become a focal point in biology and health research [1]. It continues to uncover new insights into several diseases, including cancer, depression, inflammatory intestinal disorders, neurodegenerative disorders, infectious diseases and diabetes [2].

To leverage the increasing amount of microbiome research being conducted and the data being generated, it is essential to ensure comparability between studies and improve the findability, accessibility, interoperability and reproducibility (FAIR) of microbiome data [3]. This is particularly important in resource-constrained settings where data sharing, integration and collaboration among researchers can enhance research quality and facilitate new discoveries [4–6]. To enable such integration, standardized metadata/data collection and reporting are pivotal. Indeed, the impact of biological, environmental and lifestyle factors on the composition of the microbiome has been well demonstrated, highlighting the need for comprehensive and representative metadata/data collection that is shared in an accessible and reusable fashion [7].

To address this, the Pan-African Bioinformatics Network for H3Africa (H3ABioNet) [8] created the African Human Microbiome Portal: https://microbiome.h3abionet.org/ (AHMP) [9], a web portal exclusively dedicated to metadata related to African human microbiome samples. The AHMP serves as a database for retrieving manually curated, harmonized and standardized microbiome metadata relevant to African populations. However, in the creation of this portal, difficulties were encountered in collecting and organizing research metadata due to significant variations in reporting between studies. This underscored the need for standardized metadata and data reporting protocols and tools in the microbiome field.

Currently, the leading metadata reporting standard for microbiome research is the Minimum Information for Any (x) Sequence (MIxS) standard—metagenome or environmental (MIMS) checklist developed by the Genomics Standards Consortium [10]. This standard defines a set of core descriptors for genomic and metagenomic sequences and has been adopted by public databases such as the Sequence Read Archive (SRA) and the European Nucleotide Archive (ENA). However, its implementation has been inconsistent, partly due to limited awareness, lack of practical implementation tools and inadequate data collection readiness [11].

To address these challenges, the Human Hereditary and Health in Africa (H3Africa) [12] consortium’s Microbiome Working Group [13] developed a metadata and data standardization template based on MIxS-MIMS and PhenX recommendations. PhenX is a resource for consensus measures of phenotypes and exposures. The template, known as the Microbiome Research Data Toolkit, aims to simplify metadata reporting and standardize microbiome-associated data collection. It provides recommended metadata and data attributes for research project planning, reporting and participant data standardization. The toolkit promotes research comparability, reliability and FAIR-ness with considerations for resource-constrained settings.

Standardizing microbiome data through the Microbiome Research Data Toolkit has the potential to enhance our understanding of the relationship between microbiome composition and host health as it facilitates meta-analysis and multi-omics approaches, particularly in resource-limited regions where collaborative efforts are crucial.

## Methodology

### Design and review

The Microbiome Research Data Toolkit was designed to facilitate the reporting, comparability, reusability and interoperability of microbiome study data. As such, it was subdivided accordingly with the inclusion of tools for prospective data collection, and retrospective analysis and harmonization. The criteria for the toolkit were established based on the MIxS-MIMS reporting standard, the metadata collection strategy employed to design the AHMP, literature review and feedback from researchers in the microbiome field. The collection and harmonization sections of the toolkit were designed with careful consideration of the essential phenotypes and domain-specific modules developed by the H3Africa Phenotype Harmonization Working Group, taking into account the guidance provided by PhenX [14]. Feedback received from the Microbiome Working Group played a crucial role in determining which fields should be optional or mandatory/required within these sections.

**Figure 1.**
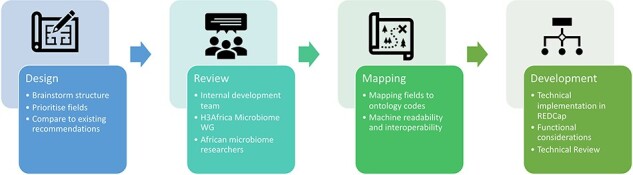
Overview of the development of the Microbiome Research Data Toolkit.

The development of the microbiome data toolkit involved several review checkpoints. The initial criteria were determined by a development task team, with the design of the technical platform reviewed by the larger Microbiome WG. Upon completion of the first draft, the toolkit was again reviewed by the Microbiome WG as well as members of the African research community, with the feedback received incorporated into the current version. The technical validity of the toolkit was also assessed by expert data managers within the H3Africa Phenotype Harmonization Working Group. The overall development process is illustrated in Fig. 1.

### Technical development

The Microbiome Research Data Toolkit was developed using Research Electronic Data Capture (REDCap), a secure web-based software platform specifically designed for research data capture [15]. REDCap offers various features to support data capture, including an intuitive interface for validated data entry, audit trails for tracking data handling and export procedures, automated export procedures for seamless data downloads to statistical packages and capabilities for data integration and interoperability with external sources.

Prior to constructing the toolkit, certain functional considerations were made to enhance its usability and accessibility within the REDCap platform. These considerations included the use of informative variable naming conventions that reflect the collected field, ensuring consistent coding throughout by including basic codes for common responses and formats, separating the toolkit into two arms to accommodate project metadata fields that are consistent across participants and participant-specific data fields that vary across participants, formatting overlapping variables between the prospective and retrospective components correctly to avoid repetition and conflicts during data collection, and maintaining a consistent visual style by utilizing only the basic REDCap forms without external modules.

While the REDCap toolkit is designed to be adaptable and allow for the inclusion of additional data elements to suit the needs of different studies, users are advised to be cautious when making modifications, considering the pre-existing branching logic within the toolkit.

### Ontology mapping

After the finalization of the toolkit, each variable within it was associated with an equivalent ontology code, whenever feasible, to enhance machine readability and enable interoperability. The application of ontology codes was carried out using the Ontology Lookup Service (OLS) [16] and Zooma [17], both developed by the European Bioinformatics Institute (EBI). Emphasis was placed on utilizing domain-specific ontologies that are well-maintained and reliable. Furthermore, a thorough review process was conducted to ensure the accuracy and correspondence of the applied codes.

## Results

**Figure 2.**
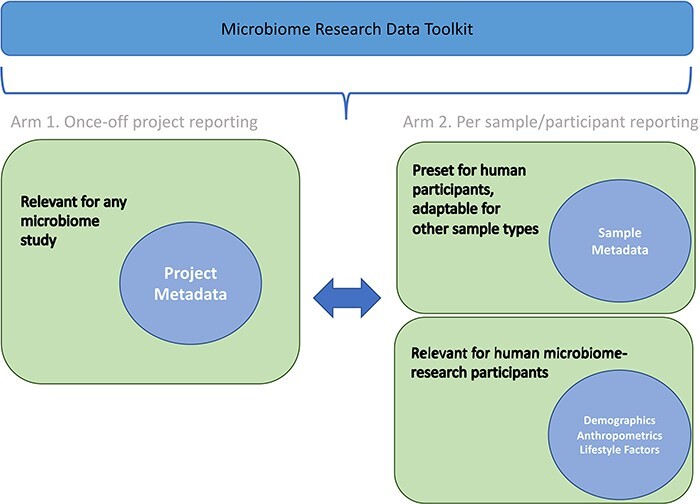
Overall structure of the Microbiome Research Data Toolkit.

The Microbiome Research Data Toolkit was officially released in September 2023. It is accessible for download and citation from various platforms, including GitHub (https://github.com/h3abionet/h3aphenstds/tree/main/Microbiome%20Toolkit%20v1.1) and figshare (https://doi.org/10.25375/uct.24218999.v2). To encourage implementation and enhancement of the toolkit, users are encouraged to provide feedback and raise issues through GitHub or by contacting the development task team (authors). The toolkit was specifically designed for multi-purpose use, including metadata reporting for study comparability and interoperability, prospective data standardization, and retrospective data harmonization to promote data interoperability and reusability. The structure of the toolkit is illustrated in Fig. 2, outlining its overall organization. By subdividing data variables, the toolkit facilitates standardized and harmonized data management for past, ongoing, and future microbiome research projects.

The toolkit comprises nine protocols, which are categorized into six sections. These protocols cover various aspects, such as project metadata, participant sample data, participant demographics, participant anthropometrics, participant lifestyle factors (both prospective and retrospective), participant alcohol consumption, smoking status and medication use (both prospective and retrospective). A summary of the sub-variables within each protocol is provided in Table 1.

**Table 1.** List of forms and data elements included in the Microbiome Research Data toolkit

Section	Criteria	Description of sub-variables
Project metadata	Project metadata	Study design, host of interest, sample type, sampling handling, sequencing, etc.
Sample metadata	Participant metadata	Case or control status, treated or untreated status, etc.
	Consent	Consent details, identifying information.
	Consent withdrawal	Withdrawal details
Demographics	Demographics	Age, sex, country of residence, language, longitude–latitude, etc.
Anthropometrics	Anthropometrics	Height, weight, fat percentage, etc.
Standardization (Prospective) component	Smoking status	Smoking history, frequency, etc.
	Alcohol consumption	Alcohol consumption history, frequency, etc.
	Medication	Detailed medication log
	Physical activity	Physical activity at work, during travel, and recreational, intensity, frequency
	Diet	Detailed diet log
Harmonization (Retrospective) Component	Smoking status	Smoking history, frequency, etc.
	Alcohol consumption	Alcohol consumption history, frequency, etc.
	Medication	Chronic medication classes
	Physical activity	Physical activity intensity, frequency
	Diet	Diet descriptions

The project metadata section of the toolkit, applicable to any microbiome research project regardless of the host being investigated, allows researchers to provide standardized information about the dataset and samples. It includes fields to describe the purpose, location, individuals involved, timing and methodology of data collection. Parameters covered in this section encompass study design, objectives, investigation type, sample and specimen types, details pertaining to sample handling (collection and storage methods), as well as DNA extraction and sequencing details (platform, amplicon region).

For research studies involving human participants, the toolkit offers data standardization and harmonization components that can be utilized based on whether the research is prospective or retrospective. The participant sample data section captures information about the samples, including anonymized sample identifiers, and provisions are made for digitizing consent associated with the sample data. Regarding participant-related biological and health data, the demographics section collects data that enables researchers to compare across different study replications. Participant anthropometrics gather valuable information concerning human body measurements. Additionally, various lifestyle factors that can influence the microbiome, such as physical activity, diet, medication, alcohol consumption and smoking status, are considered.

**Figure 3.**
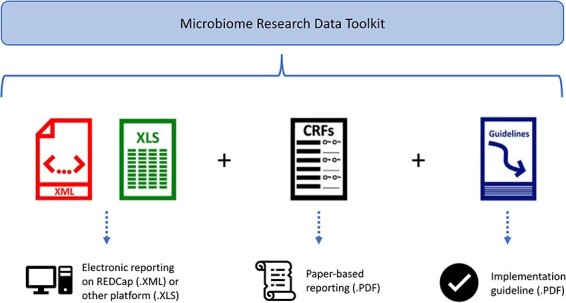
Overview of Microbiome Research Data Toolkit files.

The microbiome data standardization and harmonization toolkit, known as the Microbiome Research Data Toolkit, is available in several formats, as depicted in Fig. 3. These formats include an .XML file for implementation in the REDCap system, a data dictionary for implementation on the user’s preferred platform, a .PDF version that can serve as a Case Report Form or data collection document, and a .PDF guideline to facilitate implementation.

## Discussion

This paper introduces the Microbiome Research Data Toolkit, a versatile tool that aims to standardize microbiome research metadata, prospectively collect participant biological and lifestyle data and harmonize retrospective data. The development of the toolkit was prompted by the challenges faced in microbiome metadata and data standardization, as well as the difficulties encountered in collating content during the creation of the AHMP, a microbiome research catalog for African populations. This toolkit was created in line with the goals of the H3Africa Microbiome WG to ease the integration of microbiome components into existing research efforts. Its design incorporates basic MIxS-MIMS recommendations, relevant PhenX protocols, and FAIR data principles, with the aim of facilitating meaningful research, collaboration, meta-analyses and machine learning analysis approaches in the future.

The selection criteria for the toolkit were based on the inclusion of relevant criteria rooted in microbiome and sequencing research, while also acknowledging existing standards. The intention was not to replicate metadata reporting standards already established by the Genomics Standards Consortium but rather to create a user-friendly tool that facilitates adherence to the MIxS-MIMS standard. The project metadata section of the toolkit includes many of the recommended fields outlined by MIxS-MIMS, except for sample details, which are covered in the sample metadata section. Parameters in the project metadata section encompass study design, the host of interest and sample details such as the site sampled and sample extraction method [18]. Information on sample handling, extraction and sequencing methodologies is also requested to define the research scope and ensure comparability with other studies [19–23].

The distinction between prospective and retrospective study perspectives is crucial, as it enables or disables various forms related to participant data collection. The format of the criteria in the toolkit was informed by previous harmonization efforts within H3Africa and was based on protocols recommended by the PhenX Toolkit, which contains standard and recommended protocols for extracting valuable data in genomics research [24–26]. Sample identifiers are collected for data management purposes, while participant demographics are gathered to understand participant identities and enable comparisons across different study replications. Participant anthropometrics provide information on dietary variations between participants, as there are known correlations between individual bacteria and anthropometric, lifestyle and dietary characteristics [27–30].

A key objective for this project was to encourage utility by making it flexible, easy to use and FAIR. This was achieved in several ways. It can be employed for various purposes, and different sections can be used together or independently. The toolkit is available in different formats to accommodate user needs and implementation levels, and associated documentation provides guidance on implementation. It is adaptable to suit user requirements and is freely accessible on platforms like GitHub and figshare. The toolkit promotes interoperability through the use of ontology codes for machine-readable data exchange.

While the toolkit is adaptable and beneficial for users, there are limitations that need to be addressed. Practical implementation and validation examples are currently lacking, as the toolkit has only recently been released. Feedback and communication are encouraged to support future development and facilitate novel research collaborations. Technical capacity for electronic implementation may be limited, so a PDF version is provided to enable paper-based data collection, which is still common in low-income settings. Training materials for implementing the toolkit on technical platforms are being developed. Awareness of the toolkit needs to be raised, especially among potential users who would find immediate use cases and collaborations valuable. Moreover, research initiatives with existing cohorts may be hesitant to switch data collection methods, as it can lead to integration issues. To address this concern, the toolkit emphasizes retrospective harmonization capabilities.

In summary, we have successfully created a user-friendly toolkit for standardizing and harmonizing microbiome research data, which is applicable not only in Africa but also to a wider user community. The Microbiome Research Data Toolkit adheres to FAIR data principles, promoting data integration and interoperability. It holds significant potential for future collaborations, particularly in low-income countries where funding for omics research is limited, impacting the study power and sequencing capabilities. Robust sample sizes are crucial in omics research to ensure statistically accurate results and informed conclusions. By enabling past, present, and future microbiome research initiatives to collaborate, the toolkit facilitates novel partnerships and expedites knowledge discovery in the field. Within the H3Africa Microbiome Working Group, we plan to incorporate the toolkit in upcoming collaborative research proposals and gather feedback based on implementation experiences to further refine its development.

## Data Availability

The data underlying this article are available in the article and the included links from the article text.
